# Inactivation of Venom PLA_2_ Alleviates Myonecrosis and Facilitates Muscle Regeneration in Envenomed Mice: A Time Course Observation

**DOI:** 10.3390/molecules23081911

**Published:** 2018-07-31

**Authors:** Huixiang Xiao, Haoran Li, Denghong Zhang, Yuanyuan Li, Shimin Sun, Chunhong Huang

**Affiliations:** School of Basic Medical Sciences, Nanchang University, Nanchang 330006, China; 6300614131@email.ncu.edu.cn (H.X.); haoran.li@se14.qmul.ac.uk (H.L.); 406531816803@email.ncu.edu.cn (D.Z.); 406531817871@email.ncu.edu.cn (Y.L.); 406531815787@email.ncu.edu.cn (S.S.)

**Keywords:** varespladib, muscle regeneration, *Deinagkistrodon acutus*, fibrosis

## Abstract

Snake venom is a complex cocktail of toxins which induces a series of clinical and pathophysiological manifestations in victims, including severe local tissue damage and systemic alterations. *Deinagkistrodon acutus* (*D. acutus*) ranks among the “big four” life-threatening venomous species in China, whose venom possesses strong myotoxicity and hematotoxicity that often lead to permanent disability or muscle atrophy. Varespladib, an inhibitor of mammalian phospholipase A_2_ (PLA_2_), has been recently reproposed as an effective antidote against snakebite envenomation. The present study aimed at evaluating the protective role of varespladib on muscle regeneration in envenomed mice. Mice were grouped and subjected to inoculation with *D. acutus* venom or a mixture of venom and varespladib or control vehicle in the gastrocnemius muscle. Local injuries including hemorrhage, myonecrosis, ulceration, and systemic damages including general dysfunction, visceral failure, and inflammatory responses were observed at 1, 3, 7, 14, and 21 days. The results indicated that most of the muscle myonecrosis and hemorrhage were alleviated by varespladib. Besides, the pretreated mice recovered rapidly with lesser atrophy and muscle fibrosis. In conclusion, the findings of the present study suggested that varespladib is an effective antidote that could neutralize *D. acutus* venom and allow for earlier and improved rehabilitation outcome.

## 1. Introduction

Snakebite envenomation is an important, yet often neglected, public health concern, which causes more than 100,000 deaths and maims over 400,000 people annually, predominantly in tropical and subtropical developing countries [1]. Viperid snake envenomation contributes to the majority of ophidian incidents and is characterized by complex clinical and pathophysiological manifestations, including local tissue damage, necrosis, hemorrhage, edema, blistering, and systemic alterations such as coagulopathy, bleeding, cardiovascular shock and renal failure [1,2,3]. Victims usually present severe local tissue damage due to fast-developing necrosis at the bite site. Snake envenomation occurs most commonly in rural or remote areas where anti-venoms are not available in community settings due to their high cost, insufficient and suboptimal cold chain storage, and inadequate supply, thus leading to high morbidity and mortality [4]. Snakebites occur most frequently on the lower limbs and hands, and are characterized by necrosis of the skin, subcutaneous tissue loss and fibrosis, which can cause loss of limb function or limb amputation, both provoking permanent physical and psychological sequelae that significantly affect patients’ quality of life [5].

*Deinagkistrodon acutus* (*D. acutus*) belongs to the Viperidae family, is a medically important snake, whose venom contains a complex cocktail of toxins that affect the hemostatic system and often inflicts severe hemorrhage, edema, blistering and necrosis [6]. The biochemical toxin arsenal of *D. acutus* venom includes metalloproteinases (30.5%), C-type lectin (23.7%), serine proteases (11%), phospholipase A_2_s (PLA_2_s, 8.5%) and other proteins or peptides (26.3%) [7]. Besides the local damage, the venom also elicits systemic alterations including coagulopathy and thrombocytopenia, and can even induce a cardiovascular shock [7]. Recently, the snake venom PLA_2_ eceived substantial worldwide attention due to their extensive distribution, multiple isoenzymes, and diverse toxicities including myotoxicity, neurotoxicity, cardiotoxicity and anticoagulation, and high lipolysis activity, which often initiate the inflammatory cascade [8]. Thus, PLA_2_s are considered as promising ideal target for broad-spectrum anti-venom production [9]. Moreover, PLA_2_ exhibits synergistic effects with metalloproteinase, and PLA_2_ inhibition is correlated with increased survival of envenomed mice [10,11].

Varespladib is a nonspecific mammalian PLA_2_ inhibitor, which was initially designed as an anti-inflammatory drug, also for cardiovascular diseases [12,13]. Although failing in phase III clinical test, this small drug was identified to be highly effective in blocking venom PLA_2_s from 28 medically important snakes at nanomolar and picomolar levels [14]. Methyl varespladib, a varespladib prodrug, can be orally administrated, and it has a good chemical stability, making it a potential candidate for snakebite first-aid [15,16]. Previously, we found that varespladib could alleviate local and systemic toxicity inflicted by *D. acutus*, *Agkistrodon halys*, *Bungarus multicinctus*, and *Naja atra*, the “big four” life-threatening species in China [10]. In addition, varespladib exerts enhanced neutralization to the venom of Viperid or Elapid species who have more PLA_2_ in their venom composition. Notably, it has been also suggested that varespladib may not function as a therapeutic anti-venom to all snake venoms, particularly for snakes which have trace amount or none PLA_2_s in their total venom composition, such as *Dendroaspis polylepis* [17]. Nevertheless, our previous work was a short-term observation (6 h), while the long-term effect of varespladib on envenomed animals remains elusive. Furthermore, muscle regeneration and functional recovery are crucial criteria for developing novel therapeutic alternatives to snakebite envenomation. Therefore, the present time-course study was conducted to investigate the protective effect of varespladib on muscle regeneration. This study also evaluated the levels of serum enzyme markers and conducted the histopathological examination of the gastrocnemius muscle of mice envenomed by *D. acutus*.

## 2. Results

### 2.1. Signs of Envenomation in Mice

**Figure 1:**
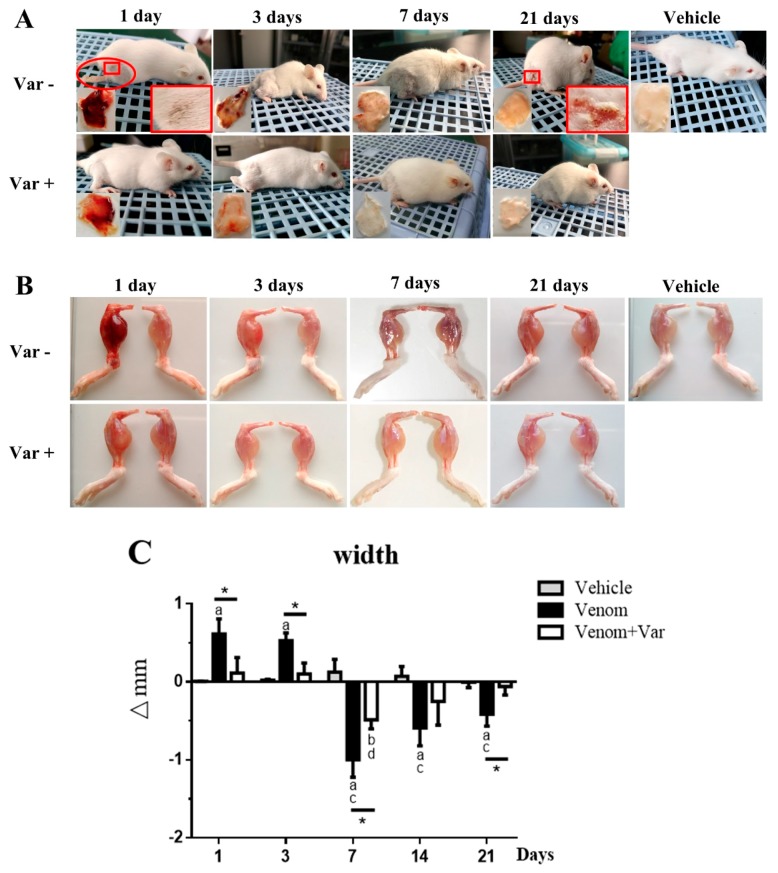
Macroscopic observation of overall signs and gastrocnemius muscle edema during the rehabilitation process. (**A**) The envenomed status of mice inoculated with *D. acutus* venom i.m. was tightly related with varespladib. Severe subcutaneous hemorrhage, paralytic hind leg, and skin ulceration developed in the venom group (day 1). Muscle contraction recovery, hemorrhage clearance, skin scabs and heals appeared serially during the rehabilitation process; (**B**) Morphological changes in gastrocnemius muscle. The envenomed muscle exhibited hemorrhage and edema during the early stage, atrophy was observed in the regenerative period; (**C**) Variation in gastrocnemius muscle width. ∆mm = width subtraction of the right limb to the left limb. Data were presented as means ± SD (*n* = 3). * *p* Venom vs. Venom + Var (*p* < 0.05), “a” *p* < 0.05 Venom vs. Vehicle, “b” *p* < 0.05 Venom + Var vs. Vehicle, “c” *p* < 0.05 Venom vs. Venom day 1, “d” *p* < 0.05 Venom + Var vs. Venom + Var day 1, Varespladib was abbreviated as Var.

At one-day post venom inoculation, mice in the venom group exhibited torpid movements, head-drooping, and somnolence. The venom inoculation resulted in severe hemorrhage extended from the gastrocnemius to the adductor medial muscle and rectus femoris muscle, and severe skin ulceration on the lateral thigh. Noticeably, mice in the venom group were paralyzed and limped; however, in the varespladib group, a significant attenuation in the hemorrhage and ulceration was observed and the mice behaved normally (Figure 1A). At day 3, the mice in the venom group partially recovered from envenomation and behaved almost normally. The hemorrhage plaque was significantly reduced and weakened, indicating that the clearance and absorption of erythrocytes might have started. However, these affected plaques and skin lesions remained yellow in appearance, similar to jaundice. Due to the interference of varespladib, at 21 day the hemorrhagic area and epidermis of the varespladib treated mice became clearer, the skin turned white, which was identical to the vehicle group. At 21 day, these envenomed mice rehabilitated to normal movement and food-intake; however, a scar persisted at the site of ulceration and the affected skin remained brown. Vehicle treated mice were healthy and active.

### 2.2. Edema and Atrophy in Gastrocnemius Muscle

The inoculated gastrocnemius muscles presented severe hemorrhage and edema (Figure 1B). The acute envenoming characteristics in gastrocnemius muscle persisted for approximately 1–3 days and gradually faded both in color and size. On the 7th day, the gastrocnemius muscle of all venom inoculated mice showed atrophy (Figure 1C). However, in the varespladib group, edema or atrophy was significantly reduced than in the venom group. The atrophy gradually turned to normality in the varespladib group; nevertheless, in the venom group mice the right limb appeared atrophic with a mean width of 0.414 mm, which was significantly thinner than the contralateral limb at day 21. In contrast, the difference in limb widths was negligible in the varespladib group. These results indicated that there was no complete recovery achieved in the venom group. Moreover, we also measured the differences in the weight of the gastrocnemius muscle during the study period (Supplementary Material Figure S1). The data showed similar trends as presented in Figure 1C. The loss in mass of envenomed muscle was difficult to be restored within 21 days.

### 2.3. Plasma Enzyme Activity

**Figure 2:**
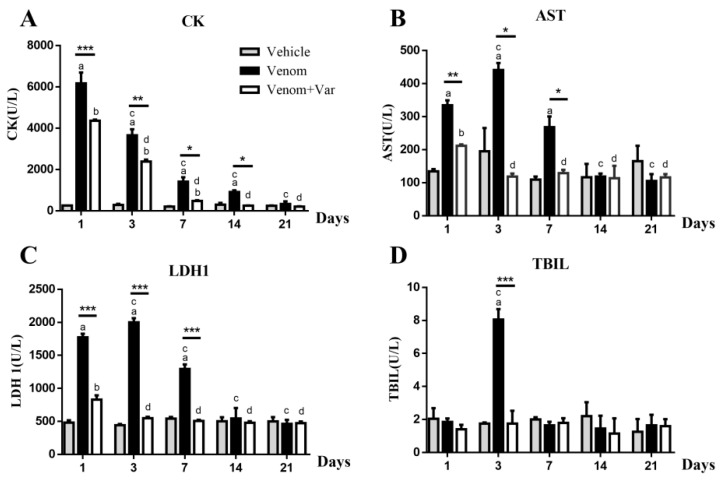
Variation in serum CK, AST, LDH1, and TBIL during a time course rehabilitation of envenomed mice. (**A**) Creatine kinase increased significantly at day 1, indicating an immediate muscle necrosis. Varespladib significantly prevented muscle degeneration; (**B**,**C**) were serum AST and LDH1 levels, significant increase occurred at the initial 7 days; (**D**) Total bilirubin, a biomarker of erythrocyte degradation, increased substantially at the 3rd day, the time of hemorrhage removal and absorption. Results were presented as means ± SD (*n* = 3). * *p* Venom vs. Venom + Var (*p* < 0.05), ** *p* < 0.01, *** *p* < 0.001,“a” *p* < 0.05 Venom vs. Vehicle, “b” *p* < 0.05 Venom + Var vs. Vehicle, “c” *p* < 0.05 Venom vs. Venom day 1, “d” *p* < 0.05 Venom + Var vs. Venom + Var day 1.

Serum creatine kinase (CK), lactate dehydrogenase isoenzyme 1 (LDH1), aspartate transferase (AST) and total bilirubin (TBIL) are the reliable biochemical indicators of myonecrosis, cardiac, and hepatic injury, respectively [18,19]. Noticeable, serum CK increase was recorded on day 1 in the venom group; the CK concentration gradually dropped with the days elapsed, and at 21 day returned to a normal level. CK values of all the time points were significantly lower in the presence of varespladib (Figure 2A).

The maximum peak value of LDH1, AST, and TBIL appeared at the third day. Significant differences in the serum values of LDH1 and AST were observed in the venom and varespladib groups from day 1 to day 7. A significant difference in the TBIL values was observed between the two groups at the 3rd day. During the following 14 days, no statistically significant difference in the LDH1, AST, and TBIL concentration among all the three groups was observed (Figure 2B–D). 

### 2.4. Histological Analysis

**Figure 3:**
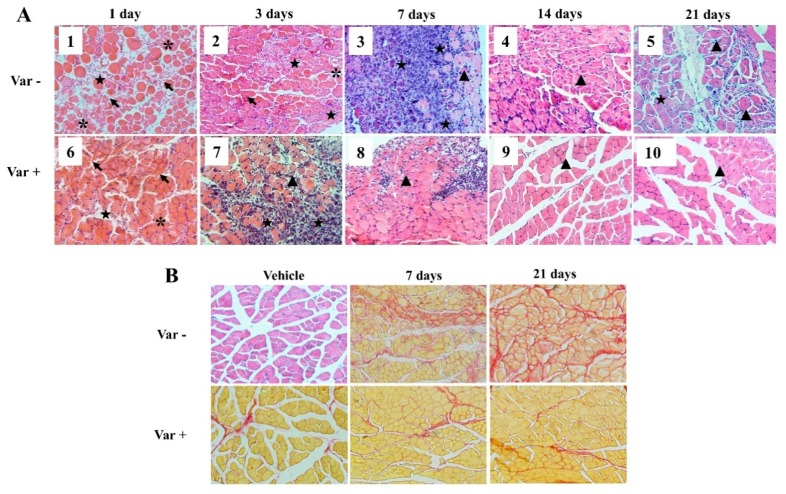
Histological examination of gastrocnemius muscle during necrosis and repair. (**A**) Hematoxylin-eosin (HE) staining of the gastrocnemius muscle. Photos of day 1 to day 3 were characterized by severe myonecrosis (asterisk), massive erythrocytes (arrow) and inflammatory cell (pentastar) infiltration. Evident basophilic myotubes were observed on day 7 (A3), and centrally-located nuclei muscle fibers (triangle) appeared simultaneously. The gastrocnemius muscle that was pretreated with varespladib showed reduced necrosis, low inflammatory reaction, better and earlier re-arrangement of myofibers; (**B**) Sirius red staining. Collagen was stained in red with a subsequent increase in its deposition accompanied by muscle regeneration. Slight fibrosis was noticed in varespladib pretreated mice. Muscle sections were examined at 200× magnification.

Consistent with the macroscopic signs, the microscopic architecture of envenomed gastrocnemius muscles was also severely damaged. Evident myonecrosis, muscular fasciae erosion, myofiber swelling and distortion were observed during the first three days (Figure 3A). Massive erythrocytes and inflammatory cell infiltration in the interspace of myofibers were also observed, which indicated the clearance of damaged erythrocytes and myocytes. These events extended beyond 7 days in the venom group but disappeared rapidly in varespladib treated mice, as shown in Figure 3(A8). After cell debris removal, the injured muscle recovered with the appearance of basophilic myotubes and centrally-located nuclei muscle fibers (Figure 3(A3)). Overall, the myonecrosis, bleeding and inflammatory infiltration was mild, and earlier regeneration occurred in varespladib treated mice (Figure 3(A6,A7)). As a result, re-arranged myocytes were formed at the 7th day (Figure 3(A8)) in mice that were pretreated with varespladib. However, the regenerated fibers in the venom group at day 21 were smaller in size as compared to the normal size fibers in the control group (Figure 3(A5)). 

The gastrocnemius section was also analyzed by Sirius red staining. Increasing collagen deposition was observed in the envenomed muscle at day 7 and day 21 (Figure 3B), which indicated the occurrence of fibrosis accompanied by muscle regeneration. In contrast, the density of collagen in the varespladib group was almost similar to the vehicle group, suggesting slight muscle fibrosis due to the intervention of varespladib.

### 2.5. Inflammation in Gastrocnemius Muscle

**Figure 4:**
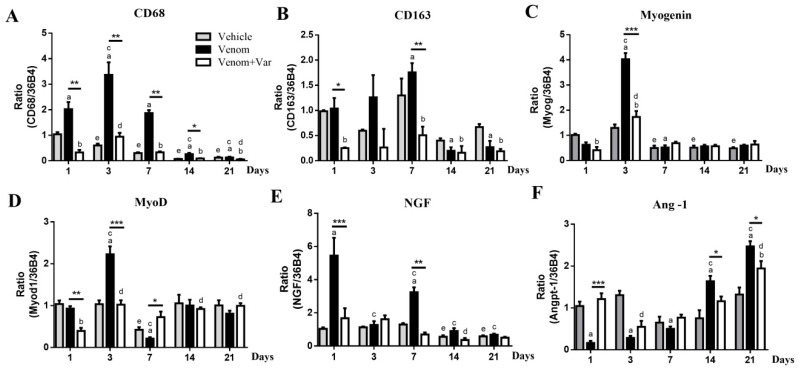
Gene expression profile of muscle regeneration. (**A**,**B**) Molecular markers of M1 and M2 macrophages, CD68 and CD163 exhibited their maximum expression at day 3 and day 7; (**C**,**D**) show the relative expression of Myogenin and MyoD, which represented the progression of muscle regeneration; (**E**) NGF expression was significantly increased in the venom group, indicating major nerve repair during the early stage of envenomation; (**F**) The bifacial variation of Ang-1 expression indicated early initiation of microvascular repair in the varespladib group, and a deferred repair in the venom group. Results were presented as means ± SD (*n* = 3). * *p*: Venom vs. Venom + Var (*p* < 0.05), ** *p* < 0.01, *** *p* < 0.001, “a” *p* < 0.05 Venom vs. Vehicle, “b” *p* < 0.05 Venom + Var vs. Vehicle, “c” *p* < 0.05 Venom vs. Venom day 1, “d” *p* < 0.05 Venom + Var vs. Venom + Var day 1, “e” *p* < 0.05 Vehicle vs. Vehicle day 1.

CD68 and CD163 are the independent specific markers of M1 and M2 macrophages responsible for necrotic debris phagocytosis and satellite cell proliferation, fusion, and differentiation, respectively [20,21]. CD68 and CD163 mRNA expressions in envenomed muscle reached their peak at the 3rd and 7th day, respectively (Figure 4A,B). CD68 and CD163 over-expression during the initial 7 days was significantly inhibited by varespladib pre-treatment. In other words, minor inflammation occurred in the varespladib group and was consistent with the histological analysis. The vehicle group also showed a slight decrease in CD68 and CD163 expression during the course, which suggested that DMSO might act as a stimulus to inflammation.

### 2.6. Coordinate Recovery in Muscle Regeneration

The coordinated recovery of muscle, microvasculature, and nerve is required for the functional reconstruction of the regenerated muscle. A significant over-expression of myoD and myogenin in the envenomed muscle was observed on day 3 (Figure 4C,D). NGF expression was also significantly up-regulated at day 1 and day 7 (Figure 4E). However, all the three genes were significantly down-regulated when the muscle was pretreated with varespladib. Interestingly, during the initial 7 days, the Ang-1 expression in the venom group was significantly lower than in the varespladib group. However, this tendency completely reversed in the subsequent period (Figure 4F).

## 3. Discussion

Snakebite envenomation is a neglected medical problem. Due to high mortality and morbidity, particularly in tropical and subtropical developing countries, snakebite envenomation has raised the concern of World Health Organization (WHO) and Médecins Sans Frontières and was reconsidered in the priority list of neglected tropical diseases (NTDs) in 2017 [22]. Consequently, significant efforts have been made in the identification of novel anti-venom drugs and complementary medicines over the recent years. Although researchers focused on the identification of new candidates, fewer concerns were paid to the muscle regeneration and rehabilitation. Irreversible damage and permanent disability often occur following snakebite envenomation if victims are not treated with antidotes timely [4]. Therefore, in this study, we established and identified the monitoring parameters for rehabilitation including envenoming signs, histopathological examination, and biomarker analysis. The study also evaluated the effect of varespladib in attenuating envenoming injuries. The 21-day observation study showed that varespladib pretreatment could prevent muscle atrophy and fibrosis, resulting in an improved tissue regeneration and function reconstruction. 

As a potent PLA_2_ inhibitor, varespladib exerted a strong suppressive effect on local injuries including hemorrhage, myonecrosis, and ulceration induced by *D. acutus* venom, and systemic damages including general dysfunction, viscera failure, and inflammatory response. These effects were attributed to the inhibition of both venom PLA_2_s and mice PLA_2_s.

Consistent with the present animal experiments and our previous findings, the envenomation with *D. acutus* venom led to severe toxic symptoms. However, the lethality was less than that of the other three venoms [10]. The envenomed mice exhibited strong adaption and self-recovery, as shown in Figure 1. The local hemorrhage in subcutis and gastrocnemius were almost disappeared, and subsequent clearance of erythrocyte triggered significant increase of total bilirubin in serum (Figure 2D) and subcutaneous heme pigment deposition similar to jaundice. Similarly, serum AST and LDH1 concentrations were also found to be at their highest level at day 3 (Figure 2B,C), which suggested that liver and heart were possibly subjected to the combined effect of the remaining toxins, hypovolemia, and transformation of bilirubin. At day 7, biochemical markers including CK, AST, LDH1, and TBIL were significantly reduced, particularly in the varespladib group; the four biomarkers reached similar values as those of vehicle mice. The swollen gastrocnemius exhibited atrophy at this time, as indicated by the complete clearance of cell debris and intercellular exudates. Furthermore, CD68 and CD163 over-expression suggested that M1 and M2 macrophages were recruited to the envenomed muscle to enhance cell debris phagocytosis, and subsequently produce growth factors for muscle regeneration [23,24].

Varespladib administration 5 min after *D. acutus* venom injection also exhibited an effective inhibitory effect on hemorrhage, myonecrosis and systemic toxicity (see Figures S2–S4). The atrophy of gastrocnemius muscle was reduced in the presence of varespladib. These results confirmed the efficacy and potential of varespladib on snakebite treatment.

Muscle regeneration is a highly orchestrated process, starting with the inflammatory response, which is characterized by macrophages infiltration, followed by the activation and proliferation of satellite cells and the differentiation to mature myofibers [25]. Satellite cells are a special type of stem cell, which are located along the surface of the myofiber under the basal lamina and maintain the ability to proliferate and differentiate to new fibers [26]. The differentiation product of satellite cells and the centrally-located nuclei muscle fibers emerged in gastrocnemius at day 7, suggesting the regeneration of injured muscle. Moreover, MyoD and myogenin are members of the myogenic regulatory factors (MRFs) family, which promotes satellite cell proliferation, fusion, and differentiation [27]. The two genes exhibited overlapping expression between 2 to 4 days after muscle damage [27,28]; however, they showed the highest expression peak at day 3.

Besides the muscle fibers, microvessel and nerve reconstruction are also crucial to functional regeneration [5,25,29]. Ang-1 is one of the major factors involved in angiogenesis and myogenesis after muscle injury [30]. Ang-1 expression was serially up-regulated during the envenoming and regeneration process, and this result was consistent with the variation described by Mofarrahi et al. [31]. Ang-1 expression in the varespladib group was significantly higher than in the venom group during the first 7 days and then dropped in the following two weeks. The delayed over-expression of Ang-1 in the venom group indicated that the microvascular recovery was a time-dependent process. Furthermore, NGF is an important neurotrophin which not only participates in peripheral nerve development and regeneration but also promotes angiogenesis [32]. In the present study, two expression peaks were observed at day 1 and day 7 in envenomed muscle. The right limb of envenomed mice was functionally paralyzed at day 1, as observed; NGF over-expression might be attributed to the nerve recovery. The second peak at day 7 was ascribed to angiogenesis; however, the underlying mechanism needs be further elucidated.

Taken together, the repair of envenomed muscle consisted of nerve recovery, phagocytosis, exudate absorption, myogenesis, and angiogenesis. However, the envenomed muscle was difficult to be restored to the original state if untreated by any antidote, and the regenerated muscle showed evident fibrosis and atrophy. Moreover, varespladib was demonstrated to be effective in diminishing the damage caused by *D. acutus* venom and thereby led to a good rehabilitation outcome in the envenomed mice. In conclusion, the findings of the present study suggested that varespladib might serve as a promising potential wide-spectrum antidote for snakebite first-aid and complementary treatment that could neutralize *D. acutus* venom effectively and achieve an earlier and improved rehabilitation outcome.

## 4. Materials and Methods

### 4.1. Venom and Varespladib

The lyophilized venom of *D. acutus* was purchased from the Huangshan Snake Farm (Huangshan, China), and stored at −20 °C before use. Varespladib was purchased from Jiyi Pharmatech Company (Shanghai, China) and dissolved in 30% dimethyl sulfoxide (DMSO) saline solution. *D. acutus* venom was also dissolved in 30% DMSO solution at the concentration of 4 mg/mL.

### 4.2. Animal Models

All animal experiments were carried out according to the Guidelines of the Laboratory Protocol of Animal Handling, and experimental protocols were approved by the Ethical Committee of Nanchang University (NDSYDWLL-201761). Male Kunming mice (25 ± 2 g) were obtained from the animal center of Nanchang University. Mice were acclimatized for seven days and were randomly divided into three groups (*n* = 15 mice/group). Hairs on the right limbs were shaved before treatment and observation. The negative control group received 50 μL vehicle (30% DMSO) in the middle of the right gastrocnemius. The venom group was treated with a similar dosage of 200 μg *D. acutus* venom. The varespladib group was treated with a mixture of *D. acutus* venom (200 μg) and varespladib that was pre-incubated at 37 °C for 10 min. The dosage of varespladib was 4 mg/kg body weight in accordance with Lewin et al. [14]. Mice were housed in a constant temperature condition (20 °C), 12 h light/dark cycle, with free access to food and water. Three mice in each group were sacrificed at 1, 3, 7, 14 and 21 days post-treatment. Besides, to explore the efficacy and potential of varespladib on snakebite first-aid treatment, additional experiments consisting of varespladib administration 5 min later after venom injection was performed (method and results see supplementary material).

### 4.3. Serum CK, AST, LDH1, and TBIL

Mice were euthanized by cervical dislocation and blood was collected by cardiac puncture. Serum was obtained by centrifugation at 12,000 rpm for 10 min at 4 °C. The enzyme activities in supernatant including creatine kinase (CK), aspartate transferase (AST), lactate dehydrogenase isoenzyme 1 (LDH1), and total bilirubin (TBIL) were determined following the manufacturer’s protocol. All kits such as CK (A032), AST (C010-1), LDH1 (A020-3) and TBIL (C019-1) were purchased from Nanjing Jiancheng Bioengineering Institute (Nanjing, China) and the values were expressed in U/L.

### 4.4. Muscle Injury and Regeneration Evaluation

Mice were routinely observed and photographed to record the overall signs of envenoming before sacrifice. Both gastrocnemius and skin on the hind limb were carefully dissected. The width and weight of envenomed and contralateral gastrocnemius muscle were measured using a vernier caliper and an electronic balance was used to evaluate the degree of edema and atrophy. The envenomed gastrocnemius was further used for RNA extraction and histological examination.

### 4.5. Quantitative Real-time PCR Analysis

Total RNA was extracted from homogenized gastrocnemius using Trizol reagent (Invitrogen, Thermo, Waltham, MA, USA), and reverse-transcribed to cDNA using a reverse transcription kit (RR047A, Takara, Kusatsu, Japan). Quantitative real-time PCR (qRT-PCR) was performed to determine the expression of CD68, CD163, myogenic differentiation (MyoD), myogenin, angiopoietin-1 (Ang-1), and nerve growth factor (NGF) in gastrocnemius muscles using GoTap qPCR Master Mix (A6001/2, Promega, Madison, WI, USA) according to the manufacturer’s protocol. The ribosome gene 36B4 was used as an endogenous control gene to normalize the expression of target genes. Primers used in the analysis are listed in Table 1. The PCR program comprised of an initial denaturation for 2 min at 95 °C, followed by 40 cycles of 95 °C or 15 s, 60 °C for 1 min and a final extension at 72 °C for 60 s. Relative gene expression was calculated using the 2^−∆∆Ct^ method.

### 4.6. Histological Analysis

Gastrocnemius muscles were fixed in 10% buffered formalin for 24 h at 4 °C, dehydrated through graded alcohols, and embedded in paraffin. The muscles were sectioned into 5 μm slices using a microtome (Leica RM2235, Wetzlar, Germany). Hematoxylin-eosin (H & E) and Sirius red (G1470, Solarbio, Beijing, China) staining were performed to examine histological alteration and collagen deposition, respectively. The procedure was performed according to the manufacturer’s protocol. Before staining, paraffin sections were deparaffinized using xylene and hydrated with distilled water. For H & E staining, section-mounted glass slides were stained in hematoxylin for 5 min and then washed in distilled water. Slides were further immersed in an eosin solution for 1 min and washed in distilled water for 10 min. After 1 min, the slides were sequentially immersed in a series of ethanol solutions (70%, 95%, and 100%), for dehydration. After immersion in xylene for 3 min twice, the slides were fixed with neutral resin and sealed with cover slip. For Sirius red staining, we used the Mayer hematoxylin for 10 min, followed by washing. Then the collagen was stained by Sirius red for 1 h. The other steps were the same as the H & E staining’s processes. Images were captured with an Olympus microscope system (IX73P2F, Olympus, Tokyo, Japan).

### 4.7. Statistical Analysis

All results are expressed as the means  ±  standard deviation (SD). Statistical comparisons were performed using analysis of variance (ANOVA), followed by ad-hoc Tukey’s test. Student’s *t*-test was used to compare the significance among different days. *p* < 0.05 was considered statistically significant. Data were statistically analyzed using the GraphPad Prism 6 software (GraphPad Software Inc., San Diego, CA, USA).

## Figures and Tables

**Table 1:** Primers used for mRNA analysis.

Gene	Sequences (5′-3′)
36B4	F: GCTTCATTGTGGGAGCAGAC
	R: TTCTCCAGAGCTGGGTTGTT
CD68	F: CCATCCTTCACGATGACACCT
	R: GGCAGGGTTATGAGTGACAGTT
CD163	F: TGTGCAGTAACGGCTGGAG
	R: ATCATGTTTGCAGTCCCAAAGA
Ang-1	F: CACATAGGGTGCAGCAACCA
	R: CGTCGTGTTCTGGAAGAATGA
NGF	F: AGACTCCACTCACCCCGTG
	R: GGCTGTGGTCTTATCTCCAAC
Myog	F: GAGACATCCCCCTATTTCTACCA
	R: GCTCAGTCCGCTCATAGCC
MyoD	F: CCACTCCGGGACATAGACTTG
	R: AAAAGCGCAGGTCTGGTGAG

## Supplementary Materials

The following supplementary materials are available online. Figure S1. Time course variation of gastrocnemius muscle weight. Figure S2. Anti-hemorrhage effect of varespladib administered after *D. acutus* envenoming. Figure S3. Variation of gastrocnemius muscle edema. Figure S4. Variation of serum CK and LDH1 at day 1, 3, 7.
