# Supplementary material for: Inactivation of Venom PLA2 Alleviates Myonecrosis and Facilitates Muscle Regeneration in Envenomed Mice: A Time Course Observation
Source: Molecules. 2018 Jul 31;23(8):1911. doi: 10.3390/molecules23081911 (PMC6222452; doi:10.3390/molecules23081911)
Supplement: Supplementary file 1 [file molecules-23-01911-s001.pdf]

Supplementary Information

Inactivation of Venom PLA_2 Alleviates Myonecrosis and Facilitates Muscle Regeneration in Envenomed Mice: A Time Course Observation

Huixiang Xiao [†], Haoran Li [†], Denghong Zhang, Yuanyuan Li, Shimin Sun and Chunhong Huang *

School of Basic Medical Sciences, Nanchang University, Nanchang 330006, China; 6300614131@email.ncu.edu.cn (H.X.); haoran.li@se14.qmul.ac.uk (H.L.); 406531816803@email.ncu.edu.cn (D.Z.); 406531817871@email.ncu.edu.cn (Y.L.); 406531815787@email.ncu.edu.cn (S.S.)

* Correspondence: chhuang@ncu.edu.cn; Tel.: +86-791-8636-1347

† These authors contributed equally to this work.

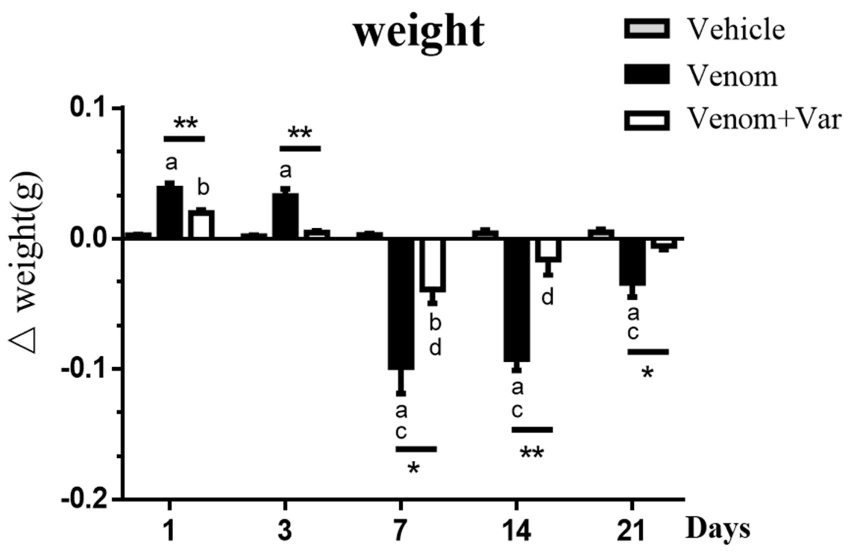

Figure S1. Time course variation of gastrocnemius muscle weight. △weight (g) = weight subtraction of right limb to left contralateral limb. The varespladib was premixed with venom before inoculation. Results were presented as means ± SD (n=3). *: Venom vs. Venom + Var (P < 0.05), **P<0.01, "a" p<0.05 Venom vs. Vehicle, "b" p<0.05 Venom + Var vs. Vehicle, "c" p<0.05 Venom vs. Venom day 1, "d" p<0.05 Venom + Var vs. Venom + Var day 1, Var was the abbreviation of varespladib.

Anti-hemorrhage effect of varespladib after envenomation

Kunming mice (25 ± 2 g, male) were randomly divided into three groups (n=3 mice/group). Mice in venom group were subjected to 100 μg *D. acutus* venom (25 μl) subcutaneously, and 50 μl 30 % DMSO was administered at the same injection site after 5 min. In the treated group, the mice were treated with 200 μg of varespladib (50 μl, dissolved in 30% DMSO) after

venom injection. Control group received saline and 30 % DMSO. Two hours later, mice were sacrificed by cervical dislocation and skinned to evaluate the anti-hemorrhage effect.

Results:

D. acutus venom generated obvious hemorrhage spot (50.75×28.54 mm) in two hours, as well ulceration and severe microvessels injury. The size and density of hemorrhage spot were evidently smaller with the treatment of varespladib (41.56×13.29 mm) (Figure S2), which indicated that varespladib had an effective anti-hemorrhage effect.

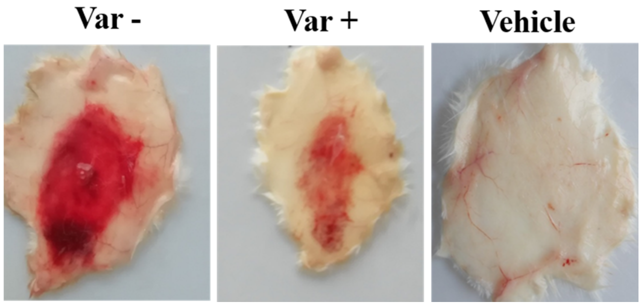

Figure S2. Anti-hemorrhage effect of varespladib administered after *D. acutus* envenoming. Smaller hemorrhage size and density was resulted by varespladib treatment.

Inhibition of myotoxicity

The same dosage of *D. acutus* venom was applied in gastrocnemius muscle of right limb, 5 min later, 50 μl 30 % DMSO or varespladib (200 μg) was injected into the envenomed muscle at the same position. The control mice were administered with saline and 30 % DMSO.

Serum CK and LDH1 level were monitored at day 1, 3, and 7 after treatment. The edema or atrophy was evaluated by weight variation of the gastrocnemius muscle.

Result:

The envenomed gastrocnemius muscle subjected to severe hemorrhage, myonecrosis and swelling, and turned to atrophy at day 7. All these signs were attenuated significantly when treated by varespladib (Figure S3). The two important serum biomarkers of local necrosis and systemic toxicity, namely, CK and LDH1 were elevated drastically at day 1, then decreased gradually in the following days (Figure S4). The level of CK and LDH1 were dramatically suppressed in varespladib treated group, and both of them were dropped to vehicle level at day 7, which suggested that varespladib was an effective alternative for first-aid of snakebite envenomation.

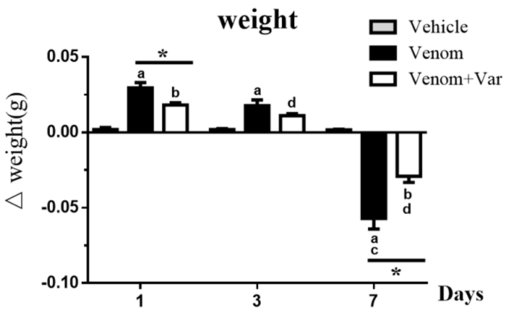

Figure S3. Variation of gastrocnemius muscle edema. △weight (g) meant the muscle weight subtraction of right limb to left limb. * Venom vs. Venom + Var (P < 0.05), "a" p<0.05 Venom vs. Vehicle, "b" p<0.05 Venom + Var vs. Vehicle, "c" p<0.05 Venom vs. Venom day 1, "d" p<0.05 Venom + Var vs. Venom + Var day 1.

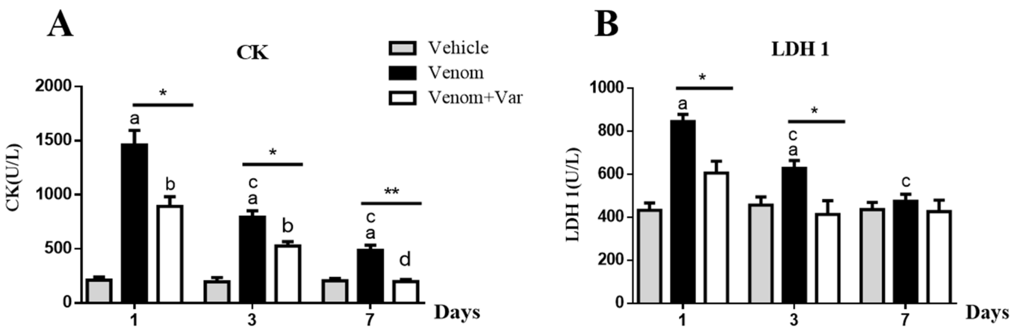

Figure S4. Variation of serum CK and LDH1 at day 1, 3, 7. Varepladib was administered at the same point of venom injection 5 mins later. CK (**A**) and LDH1 (**B**) increased dramatically at day 1 and day 3, indicating acute muscle necrosis and systemic toxicity. Both of them were decreased significantly in the presence of varespladib. * Venom vs. Venom + Var (P < 0.05), **P<0.01,"a" p<0.05 Venom vs. Vehicle, "b" p<0.05 Venom + Var vs. Vehicle, "c" p<0.05 Venom vs. Venom day 1, "d" p<0.05 Venom + Var vs. Venom + Var day 1.
